# Don’t Hold Your Breath: Indoor CO_2_ Exposure and Impaired Decision Making

**DOI:** 10.1289/ehp.120-a475a

**Published:** 2012-12-03

**Authors:** Tanya Tillett

**Affiliations:** Tanya Tillett, MA, of Durham, NC, is a staff writer/editor for *EHP*. She has been on the *EHP* staff since 2000 and has represented the journal at national and international conferences.

Elevated indoor levels of carbon dioxide (CO_2_) have been associated with impaired work/school performance, a variety of health symptoms, and poor air quality. But CO_2_ has been assumed to be simply a marker of poor ventilation rather than a detrimental exposure unto itself. Now researchers document evidence of adverse effects on adult decision-making performance associated with exposure to commonly encountered indoor levels of CO_2_, even at fixed high ventilation rates [*EHP* 120(12):1671–1677; Satish et al.].

Typical outdoor CO_2_ concentrations range from approximately 380 ppm to a high of about 500 ppm. Average indoor concentrations tend to be higher because occupants exhale CO_2_. Some studies suggest CO_2_ levels in crowded indoor spaces such as conference rooms, classrooms, and aircraft cabins can reach several thousand ppm. The American Society of Heating, Refrigerating and Air-Conditioning Engineers recommends a maximum indoor CO_2_ level of 1,000 ppm as a marker of adequate ventilation.

In the current study, researchers exposed 22 university students to CO_2_ concentrations of 600, 1,000, and 2,500 ppm in a controlled environmental chamber simulating an office environment. Participants were exposed to each concentration for 2.5 hours; all exposures occurred over the course of one day, but their sequence varied among participants. Ventilation rate, temperature, relative humidity, and other key factors were unchanged across exposures.

During each session, the participants engaged in a variety of activities, including computer-based questionnaires on perception of air quality and health symptoms. In the last 1.5 hours of each exposure, after a brief training session, they completed computer-based tests designed to analyze their decision-making performance.

The investigators observed a moderate decrease in performance for 6 of 9 decision-making measures at CO_2_ concentrations of 1,000 ppm and a more substantial decrease for 7 of 9 measures at 2,500 ppm. The authors note that the findings need to be confirmed but suggest, in a surprising turnabout, that CO_2_ should be considered an indoor pollutant, not just a proxy for other toxic pollutants. The findings also support the enforcement of current ventilation standards in buildings, and argue against reducing ventilation for the sake of energy savings.

